# Subcellular fractionation method to study endosomal trafficking of Kaposi’s sarcoma-associated herpesvirus

**DOI:** 10.1186/s13578-015-0066-2

**Published:** 2016-01-15

**Authors:** Lia R. Walker, Hosni A. M. Hussein, Shaw M. Akula

**Affiliations:** Department of Microbiology and Immunology, Brody School of Medicine at East Carolina University, Greenville, NC 27834 USA

**Keywords:** Fractionation, Ultracentrifugation, Sucrose density gradient, Endosomes, Virus entry, Endocytosis

## Abstract

**Background:**

Virus entry involves multiple steps and is a highly orchestrated process on which successful infection collectively depends. Entry processes are commonly analyzed by monitoring internalized virus particles via Western blotting, polymerase chain reaction, and imaging techniques that allow scientist to track the intracellular location of the pathogen. Such studies have provided abundant direct evidence on how viruses interact with receptor molecules on the cell surface, induce cell signaling at the point of initial contact with the cell to facilitate internalization, and exploit existing endocytic mechanisms of the cell for their ultimate infectious agenda. However, there is dearth of knowledge in regards to trafficking of a virus via endosomes. Herein, we describe an optimized laboratory procedure to isolate individual organelles during different stages of endocytosis by performing subcellular fractionation. This methodology is established using Kaposi’s sarcoma-associated herpesvirus (KSHV) infection of human foreskin fibroblast (HFF) cells as a model. With KSHV and other herpesviruses alike, envelope glycoproteins have been widely reported to physically engage target cell surface receptors, such as integrins, in interactions leading to entry and subsequent infection.

**Results:**

Subcellular fractionation was used to isolate early and late endosomes (EEs and LEs) by performing a series of centrifugations steps. Specifically, a centrifugation step post-homogenization was utilized to obtain the post-nuclear supernatant containing intact intracellular organelles in suspension. Successive fractionation via sucrose density gradient centrifugation was performed to isolate specific organelles including EEs and LEs. Intracellular KSHV trafficking was directly traced in the isolated endosomal fractions. Additionally, the subcellular fractionation approach demonstrates a key role for integrins in the endosomal trafficking of KSHV. The results obtained from fractionation studies corroborated those obtained by traditional imaging studies.

**Conclusions:**

This study is the first of its kind to employ a sucrose flotation gradient assay to map intracellular KSHV trafficking in HFF cells. We are confident that such an approach will serve as a powerful tool to directly study intracellular trafficking of a virus, signaling events occurring on endosomal membranes, and dynamics of molecular events within endosomes that are crucial for uncoating and virus escape into the cytosol.

## Background

As pathogenic hijackers of cellular machinery, viruses enter target cells via diverse, complex, and still fairly enigmatic processes that are presumed to be cell type dependent. It is widely accepted that viruses access a target cell’s interior via interactions between viral envelope glycoproteins and host cell surface receptors. Such glycoprotein: receptor interactions function in attachment and binding at the cell surface, successive internalization (uptake into the host cell), membrane fusion, and trafficking of the virus [1]. The virus entry process is generally studied by methods such as Western blotting, polymerase chain reaction (PCR), and imaging techniques using a selection of both permissive and non-permissive cells. Such studies have enlightened us as it pertains to the mechanism by which different viruses interact with receptor molecules on the surface of cells and get internalized in an effort to set up a successful infection. Several viruses, including herpesviruses utilize different modes of endocytosis to enter cells. Over the years, we have gathered scores of literature on how viruses enter cells by endocytosis primarily via the use of inhibitors of these specific pathways. However, there is dearth of knowledge as to exactly how viruses utilizing the endocytic route are trafficked within the endosomes.

Opposed to the conventional methods frequently used to assess the intracellular location of virus particles, subcellular fractionation is an advantageous approach allowing specific isolation of intact early and late endosomes. Thus, a subcellular fractionation protocol was established in our laboratory to directly study the transit of virus within the endosomes in human foreskin fibroblast (HFF) cells. Kaposi’s sarcoma-associated herpesvirus (KSHV), a *gamma*-2-herpesvirus otherwise known as human herpesvirus-8 (HHV-8), served as our model viral candidate during the standardization process, as KSHV has been demonstrated to enter HFF cells via clathrin mediated endocytosis; the most extensively studied and best characterized mechanism of endocytosis [2, 3].

KSHV has an expansive cellular tropism both in vivo and in vitro, and can infect a plethora of different cell types [4] presumably due to its ability to bind ubiquitous molecules expressed on target cells such as heparan sulfate (HS) [5]. KSHV binding to HS is thought to bring the virus in closer proximity to target cells such that perhaps more meaningful interactions with other receptor molecules, such as integrins [6], can occur to promote the actual entry process [5]. In fact, KSHV sets precedence as the first herpesvirus shown to interact with adherent target cell integrins in a step initiating the entry process [7]. Integrins are heterodimeric cell adhesion receptors composed of non-covalently associated α and β subunits [8]. To facilitate virus entry and infection, KSHV envelope associated glycoprotein B (gB) is shown to interact with integrins via its two distinct integrin recognition motifs: (1) RGD (Arg-Gly-Asp), the major integrin binding motif and minimal peptide region known to interact with subsets of integrins [7, 9]; and (2) disintegrin-like domain (DLD), the lesser studied and highly conserved integrin recognition motif that binds integrins RGD-independently [10–12]. A multitude of studies have implicated KSHV gB interactions with RGD-binding integrins, α3β1, αVβ3, and αVβ5, as valuable for infectious virus entry [7, 13–15]. However, both RGD and non-RGD-binding integrins are believed to aid equally in virus entry [16]. For instance, recent studies by us [10] identified KSHV gB interactions with cell surface expressed integrin α9β1, a DLD-binding integrin, crucial to promoting viral infection of cells [10]. Aside from integrins, other receptors shown to have a role in KSHV entry are ephrin receptor tyrosine kinase A2 (EphA2) [17], dendritic cell-specific intercellular adhesion molecule-3-grabbing non-integrin (DC-SIGN) [18], and human cystine/glutamate exchange transporter system x–c (xCT) [1]. After binding to receptors (i.e. proteins, carbohydrates, or lipids), the vast majority of viruses utilize endocytic routes to enter target cells [19].

The majority of our current knowledge as it relates to virus entry has been extrapolated qualitatively by means of electron microscopy and other imaging techniques. Alternatively, here we sought to map KSHV trafficking via the endosomes using biochemical analysis. In the process, our study defines a critical role for α9β1 integrin in the ability of KSHV to escape LEs of HFF cells.

## Results

### KSHV particles are trafficked beyond the early endosome for late endosomal escape

**Fig. 1:**
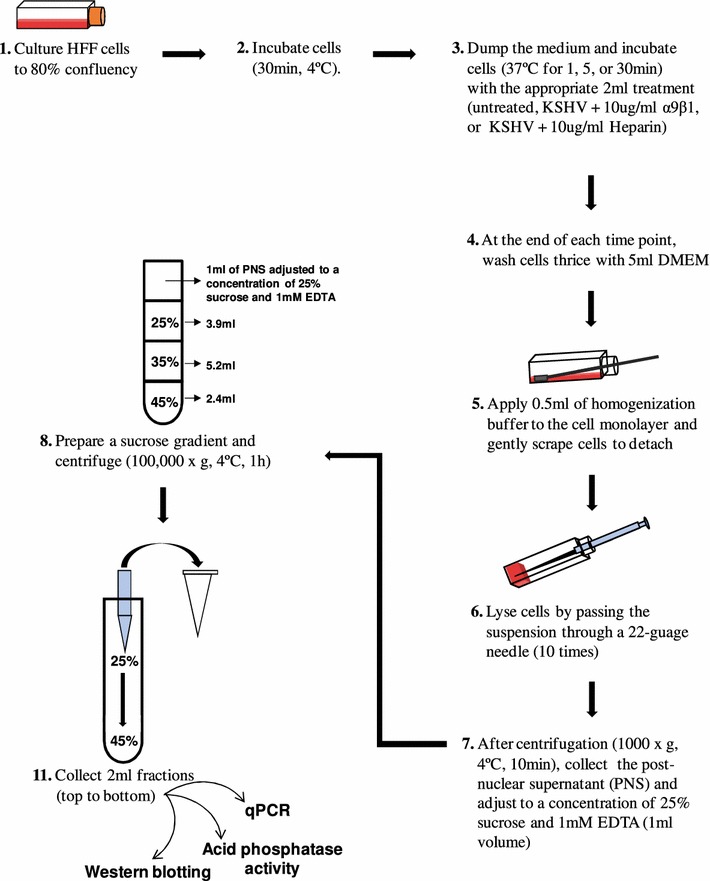
Schematic representation of the sucrose flotation assay for endosome purification. This study used uninfected and KSHV (MOI of 5 DNA copies/cell) infected HFF cells

KSHV enters HFF cells via clathrin-mediated endocytosis [20]. Typically, cargo internalized by this mechanism of endocytosis is thought to be directed from early endosomes (EEs) to late endosomes (LEs) then lysosomes to undergo degradation or changes to conformation [21]. To begin with, untreated HFF cells grown to 80 % confluency were cooled (incubated at 30 min at 4 °C), incubated with DMEM alone (30 min at 37 °C), collected and lysed in homogenization buffer; after which, centrifugation was performed to derive the post-nuclear supernatant (PNS) containing intracellular organelles in suspension (Fig. 1). The PNS adjusted to a concentration of 25 % sucrose and 1 mM EDTA was then subjected to fractionation via sucrose density gradient centrifugation followed by fraction collection (Fig. 1).

**Fig. 2:**
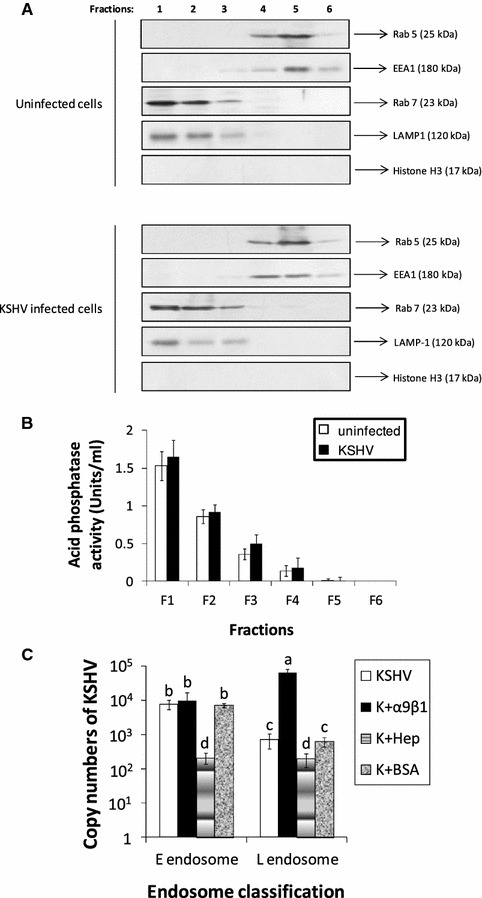
Detection of KSHV in endosomal fractions. **A** Distribution of endosomal membranes was determined from uninfected and KSHV infected HFF cells using sucrose gradient fractions. Equal-volume aliquots from each gradient fraction were separated by SDS-PAGE and probed by Western blotting against organelle markers: Rab5 and EEA1 (EE markers), Rab7 (LE marker), LAMP1 (LE and lysosome marker), and histone H3 (nuclear marker). **B** Lysosome detection in sucrose gradient fractions of uninfected and KSHV infected HFF cells. Acid phosphatase activity was analyzed by using Acid Phosphatase Assay Kit (Sigma). **C** KSHV escapes from the LE. Following gradient centrifugation, viral genomic DNA was extracted from each recovered fraction. Fractions were then quantified for the content of internalized viral DNA by performing qPCR using specific primers. *Data* (*panels* **B**, **C**) represent the average ±SD (*error bars*) of three experiments. *Columns* with different alphabets indicate statistical significance (p < 0.05) by least significant difference (LSD)

Collected gradient fractions 1–6 of untreated HFF cells were analyzed by SDS-PAGE and Western blotting for the distribution of organelle markers: Rab7 (LE marker), Rab5 and early endosome antigen 1 (EEA1; EE markers), and lysosome-associated membrane protein (LAMP1; LE and lysosome marker) [22, 23] (Fig. 2A). Fractions 1–3 contained the LEs with the majority being concentrated in fraction 1 as detected by Rab7 and LAMP1 expression. Fractions 4–6 contained the EEs with the majority being concentrated in fraction 5 as detected by Rab5 and EEA1 expression (Fig. 2A). To exclude possible contamination of the fractions with other cellular components, nuclear marker histone H3 [24] served as a negative control (Fig. 2A); gradient fractions are void of nuclei which are totally removed during the post-homogenization centrifugation step [25]. Notably, comparatively assessing the density profile of collected gradient fractions 1–6 from both uninfected and KSHV infected HFF cells by refractometric analysis revealed that KSHV infection of HFF cells did not alter floating density of the endosome containing gradient fractions (data not shown), nor did KSHV infection of HFF cells seem to alter fraction distribution of early and late endosomes (Fig. 2A). LEs (sometimes referred to as pre-lysosomes [26], lysosomes, and the endolysosome hybrid organelle—from where mature lysosomes bud—are said to be difficult to distinguish [27], as basic sucrose gradient centrifugation procedures are shown to forgo total separation of such organelles [28]. Here, acid phosphatase (an established enzyme marker for identifying lysosomes in subcellular fractions; [29]) activity was analyzed to detect the level of lysosome enrichment in collected gradient fractions (Fig. 2B). Upon fraction comparison, acid phosphatase activity in both the uninfected and KSHV infected HFF cells was detected in fractions 1–3 with fraction 1 displaying the highest phosphatase activity. Activity of acid phosphatase foreseeably coincided with the LE fractions (fractions 1–3; Fig. 2A); although acid hydrolases (e.g. acid phosphatase) majorly localize in lysosomes, these enzymes can be found in LEs as well [27]. Therefore, such findings provide even further evidence for the presence of LEs in the said fractions.

Having established the fractions that contain early and late endosomes, we purified endosomes via a sucrose flotation gradient to quantitatively track KSHV in infected HFF cells post-internalization and to characterize possible role(s) for DLD-binding integrins during this stage of the entry process via qPCR (Figs. 1, 2C). Earlier studies described KSHV entry as a rapid process wherein the capsid delivers the genetic material to the nucleus allowing eventual expression of viral transcripts in as early as 30 min post infection (PI) [30, 31]. Here, we monitored KSHV particles in endosomes 30 min PI. In the case of cells infected with KSHV, there was notably more KSHV DNA detected in EE containing fractions compared to the fractions containing LEs (Fig. 2C). Similar results were observed in cells infected with a mixture of KSHV and BSA (Fig. 2C). Though incubating soluble integrin α9β1 with KSHV did not significantly alter the levels of KSHV in EEs, it significantly impeded the ability of KSHV to escape the LEs (Fig. 2C). These results suggest a possible role for α9β1 integrin in virus-mediated endosomal escape. Additionally, infection in the presence of our positive control, heparin, results in a significant drop in the number of virus particles observed in both EE and LE containing fractions (Fig. 2C). Notably, pre-treatment of KSHV with soluble heparin is shown to impede the initial attachment of the virus to HS on the cell surface [32], thus blocking binding, signal induction, entry, and efficient infection of target cells [5, 33]. Based on these results, as it pertains to HFF cells, we presume KSHV to be a late penetrating virus that exits from the LE into the cytosol. We could not detect HSV-2 in either the early or late endosome (data not shown), as earlier studies demonstrated HSV-2 to enter target cells via fusion at the cell membrane [20].

**Fig. 3:**
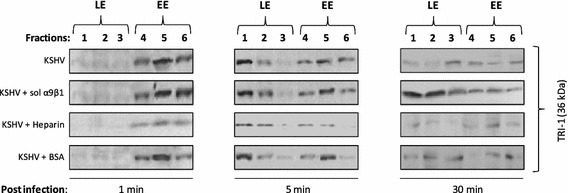
Confirming the presence of KSHV in sucrose gradient fractions at different early time points. Fractions (5 µg protein) collected at different early time points (1, 5, or 30 min) during the course of infection were resolved by SDS-PAGE followed by Western blot analysis with anti-KSHV TRI-1 antibodies

To further confirm the presence of KSHV in endosome containing gradient fractions, fractions collected at different early time points (1, 5, or 30 min) during the course of infection were resolved by SDS-PAGE and subsequently Western blotted with antibodies directed against minor capsid protein KSHV ORF62-encoded triplex component I (TRI-1) for detecting the presence of viral capsids (Fig. 3). Expression of TRI-1 is ideal for detection of the virus, as the virus envelope is lost upon fusion/penetration. At 1 min post KSHV infection, significantly more virus was observed in EE containing fractions 4–6 versus LE fractions with most virus being detected in fraction 5 (Fig. 3). Similar patterns were observed at 1 min PI when the virus was incubated with BSA or soluble α9β1 (Fig. 3). Incubating KSHV with heparin lowered the percentage of virus being internalized as detected by limited staining for TRI-1 in EEs (Fig. 3). By 5 min PI, a significant portion of the virus had reached LEs in KSHV infected cells or cells infected with a mixture of KSHV that was incubated with BSA or α9β1 (Fig. 3). Heparin blocked KSHV internalization effectively as described at 1 min PI. By 30 min PI, there was a marked drop in the levels of virus in LEs (especially LE fraction 1) in cells that were infected with KSHV or a mixture of KSHV and BSA (Fig. 3). Surprisingly, we found an increase in the levels of virus associated with LEs of cells that were infected with a mixture of KSHV and soluble α9β1. Overall, Western blotting results monitoring TRI-1 expression as an indicator of KSHV in collected gradient fractions of virus infected HFF cells (Fig. 3) mimic qPCR results (Fig. 2C), thus confirming escape of KSHV from the LE to the cytoplasm in HFF cells.

### Immunofluorescence microscopy demonstrates KSHV to localize in EEs and LEs

**Fig. 4:**
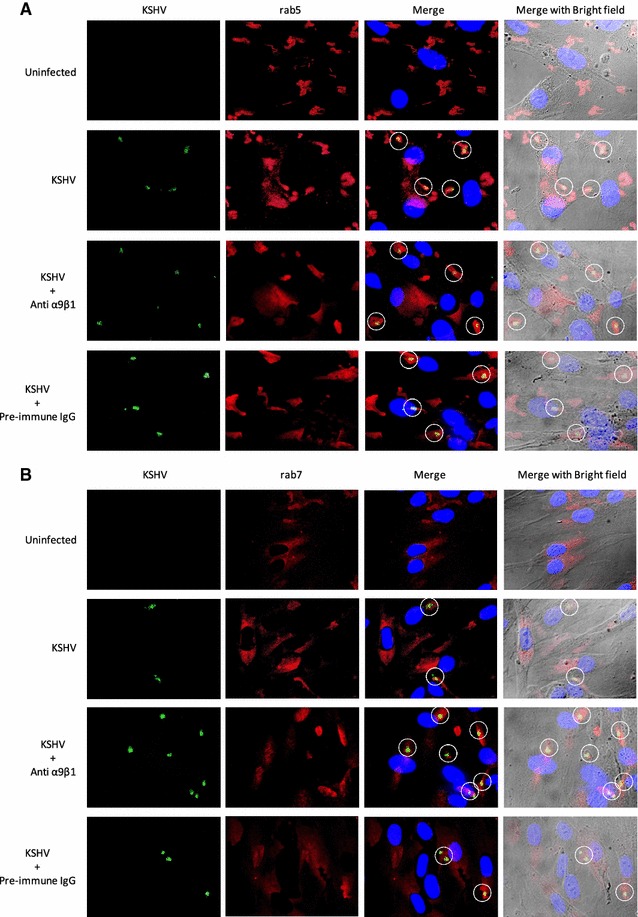
Antibodies to integrin α9β1 inhibit KSHV escape from the late endosome. **A** KSHV is present in Rab5-positive EEs and Rab7-postive LEs. HFF cells were either uninfected or pre-treated (1 h at 37 °C) with antibodies (20 µg/ml) to α9β1 or pre-immune IgG prior to infection with FITC-KSHV for 1 min (**A**) and 30 min (**B**) at 37 °C. Cells were washed, fixed, and subsequently immunostained with TRITC labeled anti-Rab5 antibodies (**A**) and TRITC labeled anti-Rab7 antibodies (**B **) prior to mounting using an anti-fade reagent containing DAPI and examining under a fluorescent microscope. Representative images are provided at ×1000 magnification. The *white circles* indicate co-localization of FITC-KSHV with the respective endosome marker within the cell (*panels* **A** and **B**)

**Fig. 5:**
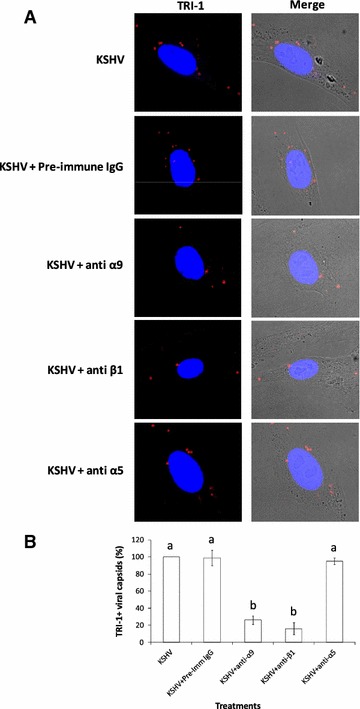
Antibodies against α9 and β1 integrin subunits block KSHV nucleocapsid trafficking to the perinuclear region by 30 min PI. **A** HFF cells were untreated or pre-treated (1 h at 37 °C) with antibodies (20 µg/ml) to α9, β1, α5 or a pre-immune IgG prior to infection with KSHV for 30 min at 37 °C. Cells were washed, fixed, and subsequently immunostained for TRI-1 minor capsid protein. Representative fluorescence imaging depicting TRI-1 positive viral capsids in infected HFF cells are shown. Mag ×1000. **B** Statistical analysis on the viral capsids that accumulated around the perinuclear region was determined based on randomly counting the number of TRI-1 positive KSHV capsids around 10 different nuclei. The TRI-1 positive viral capsids around the perinuclear region for different treatments is presented as percentage (%) as compared to cells infected with KSHV alone (n = 60). *Data* represent the average ±SD (*error bars*) of three experiments. *Columns* with different alphabets are statistically significant (p < 0.05) by LSD

For further confirmation, we employed the traditional method of immunofluorescence imaging with FITC-KSHV and TRITC labeled antibodies to Rab5 and Rab7 to monitor the trafficking of KSHV into the target cells. By as early as 1 min PI of cells, KSHV was associated with EEs as observed by co-localization of the FITC-KSHV with Rab5 expression (Fig. 4a). Treating cells with antibodies to α9β1 or pre-immune IgGs did not significantly alter this co-localization (Fig. 4a). At 30 min PI, a fraction of KSHV was associated with LEs as observed by co-localization of the FITC-KSHV with Rab7 expression (Fig. 4b). A similar result was observed when the cells were treated with pre-immune IgGs (Fig. 4b). However, the number of co-localizing events substantially increased when the cells were treated with antibodies to α9β1 prior to infection (Fig. 4b). For further authentication, subsequent intracellular trafficking of KSHV upon cytosolic delivery (post-late endosomal penetration) was similarly analyzed via immunofluorescence microscopy using KSHV and antibodies directed against minor capsid protein TRI-1 (Fig. 5). By 30 min PI, KSHV was shown to accumulate at the perinuclear region as observed by TRI-1 positive viral capsids (similar results were observed in cells treated with pre-immune IgGs) (Fig. 5A, B). However, when cells were treated with antibodies against integrin subunits (α9 or β1), a substantial reduction in perinuclear TRI-1 expression corresponding to KSHV nucleocapsids was observed (Fig. 5A, B). This reduction in the number of capsids positive for TRI-1 was not observed when the cells were treated with antibodies to integrin subunit α5 (Fig. 5A, B). Anti-integrin α5 antibodies which have no effect on KSHV entry and infection [10, 14] served as a negative control (Fig. 5A, B). Thus, these results clearly denote the ability of antibodies against integrin subunits (α9 or β1) to block virus infection by preventing the escape of KSHV from LEs. Overall, we conclude virus:α9β1 interactions to play a key role in trafficking of KSHV via endosomes to eventually escape into the cytoplasm.

## Discussion

Receptors are considered necessary cell surface molecules instrumental for successful virus infection [34]. Herpesvirus entry occurs via viral glycoprotein engagement of target cell receptor molecules. Those receptors considered valuable for KSHV entry into HFF cells are binding receptor, HS, RGD-binding integrins (α3β1, αVβ3, αVβ5; [5, 7, 14, 35]), and DLD-binding α9β1 integrin [10]. In particular, integrins are known to alter different stages of the virus entry process. Much work has been done to establish a role for RGD-binding integrins in regulating KSHV infectious entry. Specifically, RGD of KSHV gB functionally interacts with integrins α3β1, αVβ3, and αVβ5 that have a role in the initial internalization of the virus [5, 7, 14, 35]. However, very little is known about the role of DLD-binding integrins during the initial stages of KSHV infection. Employing subcellular fractionation to assess endosomal trafficking of KSHV, the present study, for the first time, deciphers a role for DLD-binding α9β1 integrin in regulating KSHV entry.

This study utilized a sucrose flotation gradient assay to track endocytosed viral cargo through the EEs and LEs via subcellular fractionation (Fig. 1). Determining at which interface a particular membrane will be detected depends on the lipid to protein content ratio. Endosomal membranes are considered low density and lipid-rich [25]. LEs were expected to be recovered from the interface between 8 and 25 % sucrose, whereas the EEs were expected between 25 and 35 % sucrose [36, 37].

In the present report, the PNS derived from KSHV infected HFF cells untreated or treated with soluble α9β1 heparin, or BSA was subjected to high-speed sucrose density gradient centrifugation (Fig. 1). Mutually, qPCR and Western blotting results revealed significantly lower levels of KSHV in LE fractions obtained from KSHV infected cells 30 min PI compared to cells that were infected with KSHV incubated in the presence of soluble α9β1 (Figs. 2C, 3). In other words, incubating KSHV with soluble α9β1 blocked the escape of KSHV from LEs to cytoplasm. Taken together, the results from the use of subcellular fractionation experiments proved the following: (1) DLD-binding integrin α9β1 plays a crucial role in the trafficking of KSHV via endosomes; (2) KSHV internalization into EEs is a rapid event (as little as 1 min PI); and (3) KSHV capsids exit LEs to generate a successful infection.

This study confirmed the results from the subcellular fractionation experiments by also utilizing traditional immunofluorescence microscopy (Figs. 4, 5). These findings have taken our current knowledge of KSHV entry a step further, delineating yet another dynamic role for integrins in a post-internalization stage of KSHV infection of HFF cells. Notably, a previous study by Schornberg et al. also provides evidence that viruses utilize integrins for regulation of virus entry at steps beyond binding and initial internalization to promote virus penetration from endocytic organelles. Specifically, they suggest that cell surface integrin expression mediates virus entry at this post-internalization step via controlling endosomal cathepsins (proteases found in endosomes that mediate intracellular proteolysis; [38, 39]). With further studies, we seek to resolve: (1) the role for α9β1 in mediating proteolytic activity within endosomes; and (2) the link between post-internalization virus:integrin interactions and endosomal acidification in supporting a successful virus infection of cells.

## Conclusions

Subcellular fractionation is a powerful tool to analyze the biology of virus entry—especially for those viruses that enter via endocytosis. Unlike conventional methods, this tool offers better maneuverability for appreciating events occurring within individual intracellular organelles with respect to the internalized virus. The only major problem with this approach is that it may seem laborious compared to conducting immunofluorescence or inhibitor-based infection assays. However, the benefits are very many, as subcellular fractionation is a privileged technique that can be optimized to analyze: (1) signaling events occurring on the surface of endosomes critical to the transit of virus particles; (2) events on the endosomal membrane eventually resulting in the escape of the capsid; (3) the endosomal milieu critical for the trafficking of the virus; (4) conformational changes on the viral envelope and capsid that support successful transit via endosomes; and (5) real-time trafficking of the virus particle through the intracellular components.

## Methods

### Cells

HFF cells were propagated as per standard laboratory protocols [40].

### Antibodies

Anti-integrin α9 (H-198) antibodies (Santa Cruz Biotechnology); anti-integrin α5 (P1D6), β1 (6S6), α9β1 (Y9A2; Millipore) antibodies; Rab5 and Rab7 (D95F2) XP™ antibodies (Cell Signaling Technology); purified mouse anti-EEA1 (BD Transduction Laboratories) antibody; anti-LAMP1 antibody and Anti-Histone H3 antibody (Abcam); mouse monoclonal antibody (5B7B6) to KSHV *orf62* encoded minor capsid protein, TRI-1 (Thermo Scientific) were used in this study.

### Proteins and reagents

Recombinant human integrin α9β1 (R and D Systems); heparin, and fluorescein isothiocyanate (FITC) (Sigma) were used in this study.

### Sucrose flotation gradient

Confluent monolayers of adherent HFF cells were cooled (4 °C for 30 min) and either remained uninfected (undergoing incubation at 37 °C for 30 min) or were infected (undergoing incubation 37 °C for 1, 5, or 30 min) with wild type KSHV (MOI of 5 DNA copies/cell) in the presence of DMEM only or 10 µg/ml α9β1, BSA, or heparin. After the designated time point, cells were washed thrice with DMEM followed by the application of 0.5 ml of homogenization buffer (250 mM sucrose, 1 mM EDTA, 1 mM phenylmethylsulfonyl fluoride (PMSF)), in which cells were gently detached using a cell scraper, lysed, and further processed for examination by a sucrose flotation assay as previously described [36]. Specifically, after centrifugation (1000×*g*), the PNS was collected and adjusted to a concentration of 25 % sucrose and 1 mM EDTA in 1 ml total volume. In 1 ml increments, 2.4 ml of 45 % sucrose was transferred to the bottom of a SW41Ti tube and successively overlaid with 5.2 ml of 35 % sucrose, 3.9 ml of 25 % sucrose, and 1 ml of PNS in 25 % sucrose. Following centrifugation (100,000×*g*), 2 ml fractions were collected from top to bottom, and densities were measured by refractometry. These fractions were further analyzed using endosomal markers and KSHV. Herpes simplex virus-2 (HSV-2) was used as control in this study.

### Western blotting and acid phosphatase activity assay

Western blotting was performed as per earlier studies [30] to monitor expression of Rab 5, EEA1, Rab7, LAMP1, and KSHV TRI-1. Acid phosphatase activity for lysosome identification was analyzed in 50 µl sample from each fraction by using Acid Phosphatase Assay Kit (Sigma) according to the manufacturer’s instructions.

### Monitoring KSHV levels in gradient fractions by qPCR

KSHV and HSV-2 levels in different fractions collected after sucrose flotation gradient centrifugation were determined by isolating total genomic DNA prior to monitoring *orf50* [41] and UL5 gene copies [42], respectively, by qPCR [41]. As a benchmark for successful infection, *orf50* was monitored as per early studies; *orf50* is said to be expressed within 30 min of successful KSHV infection [30].

#### Immunofluorescence microscopy

 FITC-KSHV and FITC-HSV-2 were generated as per earlier protocols [20]. In order to map the endosomal location of KSHV, HFF cells (75 % confluent) cultured in 8 well chamber slides were either uninfected or infected with FITC-KSHV for 1 or 30 min at 37 °C. Post-infection, cells were washed in phosphate-buffered saline (PBS) and fixed with 3.7 % formaldehyde in PBS for 10 min. After fixing, cells were washed, permeabilized using 0.1 % Triton X-100 in PBS for 3 min, washed again, and incubated for 20 min at room temperature with PBS containing 1 % bovine serum albumin (BSA) to block non-specific binding sites. Cells were then incubated successfully (1 h at 37 °C) with the appropriate primary (anti-Rab5, or anti-Rab7) and secondary (goat anti-rabbit TRITC) antibodies. Immunostained cells were washed in PBS and imaged with a Nikon fluorescent microscope using appropriate filters. To study the escape of KSHV from the LEs, we infected cells with KSHV for 30 min, fixed the cells as described above, and sequentially stained with anti-KSHV TRI-1 antibodies and goat anti-mouse TRITC antibodies prior to examining under a fluorescent microscope.

## Abbreviations

KSHV: Kaposi’s sarcoma-associated herpesvirus
EE: early endosome
LE: late endosome
DLD: disintegrin-like domain
gB: glycoprotein B
PI: post infection
HFF: human foreskin fibroblast
PNS: post-nuclear supernatant

## References

[1] Zhang W, Gao SJ (2012). Exploitation of cellular cytoskeletons and signaling pathways for cell entry by Kaposi’s sarcoma-associated herpesvirus and the closely related rhesus rhadinovirus. Pathogens.

[2] Bhattacharyya S, Warfield KL, Ruthel G, Bavari S, Aman MJ, Hope TJ (2010). Ebola virus uses clathrin-mediated endocytosis as an entry pathway. Virology.

[3] Hernaez B, Alonso C (2010). Dynamin- and clathrin-dependent endocytosis in African swine fever virus entry. J Virol.

[4] Hertel L (2011). Herpesviruses and intermediate filaments: close encounters with the third type. Viruses.

[5] Akula SM, Wang FZ, Vieira J, Chandran B (2001). Human herpesvirus 8 interaction with target cells involves heparan sulfate. Virology.

[6] Barczyk M, Carracedo S, Gullberg D (2010). Integrins. Cell Tissue Res.

[7] Akula SM, Pramod NP, Wang FZ, Chandran B (2002). Integrin α3β1 (CD 49c/29) is a cellular receptor for Kaposi’s sarcoma-associated herpesvirus (KSHV/HHV-8) entry into the target cells. Cell.

[8] Giancotti FG, Ruoslahti E (1999). Integrin signaling. Science.

[9] Wang FZ, Akula SM, Sharma-Walia N, Zeng L, Chandran B (2003). Human herpesvirus 8 envelope glycoprotein B mediates cell adhesion via its RGD sequence. J Virol.

[10] Walker LR, Hussein HA, Akula SM (2014). Disintegrin-like domain of glycoprotein B regulates Kaposi’s sarcoma-associated herpesvirus infection of cells. J Gen Virol.

[11] Eto K, Huet C, Tarui T, Kupriyanov S, Liu HZ, Puzon-McLaughlin W (2002). Functional classification of ADAMs based on a conserved motif for binding to integrin alpha 9beta 1: implications for sperm-egg binding and other cell interactions. J Biol Chem.

[12] Feire AL, Roy RM, Manley K, Compton T (2010). The glycoprotein B disintegrin-like domain binds β 1 integrin to mediate cytomegalovirus entry. J Virol.

[13] Garrigues HJ, Rubinchikova YE, Dipersio CM, Rose TM (2008). Integrin αVβ3 Binds to the RGD motif of glycoprotein B of Kaposi’s sarcoma-associated herpesvirus and functions as an RGD-dependent entry receptor. J Virol.

[14] Veettil MV, Sadagopan S, Sharma-Walia N, Wang FZ, Raghu H, Varga L (2008). Kaposi’s sarcoma-associated herpesvirus forms a multimolecular complex of integrins (αVβ5, αVβ3, and α3β1) and CD98-xCT during infection of human dermal microvascular endothelial cells, and CD98-xCT is essential for the postentry stage of infection. J Virol.

[15] Chandran B (2010). Early events in Kaposi’s sarcoma-associated herpesvirus infection of target cells. J Virol.

[16] Hussein HA, Walker LR, Abdel-Raouf UM, Desouky SA, Montasser AK, Akula SM (2015). Beyond RGD: virus interactions with integrins. Arch Virol.

[17] Hahn AS, Kaufmann JK, Wies E, Naschberger E, Panteleev-Ivlev J, Schmidt K (2012). The ephrin receptor tyrosine kinase A2 is a cellular receptor for Kaposi’s sarcoma-associated herpesvirus. Nat Med.

[18] Rappocciolo G, Jenkins FJ, Hensler HR, Piazza P, Jais M, Borowski L (2006). DC-SIGN is a receptor for human herpesvirus 8 on dendritic cells and macrophages. J Immunol.

[19] Yamauchi Y, Helenius A (2013). Virus entry at a glance. J Cell Sci.

[20] Akula SM, Naranatt PP, Walia NS, Wang FZ, Fegley B, Chandran B (2003). Kaposi’s sarcoma-associated herpesvirus (human herpesvirus 8) infection of human fibroblast cells occurs through endocytosis. J Virol.

[21] Vonderheit A, Helenius A (2005). Rab7 associates with early endosomes to mediate sorting and transport of Semliki forest virus to late endosomes. PLoS Biol.

[22] Harley CA, Dasgupta A, Wilson DW (2001). Characterization of herpes simplex virus-containing organelles by subcellular fractionation: role for organelle acidification in assembly of infectious particles. J Virol.

[23] Wolf M, Deal EM, Greenberg HB (2012). Rhesus rotavirus trafficking during entry into MA104 cells is restricted to the early endosome compartment. J Virol.

[24] Iwahara T, Bonasio R, Narendra V, Reinberg D (2012). SIRT3 functions in the nucleus in the control of stress-related gene expression. Mol Cell Biol.

[25] Huber LA, Pfaller K, Vietor I (2003). Organelle proteomics: implications for subcellular fractionation in proteomics. Circ Res.

[26] Braulke T, Bonifacino JS (2009). Sorting of lysosomal proteins. Biochim Biophys Acta.

[27] Repnik U, Cesen MH, Turk B (2013). The endolysosomal system in cell death and survival. Cold Spring Harb Perspect Biol.

[28] Waugh MG, Chu KM, Clayton EL, Minogue S, Hsuan JJ (2011). Detergent-free isolation and characterization of cholesterol-rich membrane domains from trans-Golgi network vesicles. J Lipid Res.

[29] Zhao H, Ruberu K, Li H, Garner B (2013). Analysis of subcellular [57Co] cobalamin distribution in SH-SY5Y neurons and brain tissue. J Neurosci Methods.

[30] Dyson OF, Traylen CM, Akula SM (2010). Cell membrane-bound Kaposi’s sarcoma-associated herpesvirus-encoded glycoprotein B promotes virus latency by regulating expression of cellular Egr-1. J Biol Chem.

[31] Krishnan HH, Naranatt PP, Smith MS, Zeng L, Bloomer C, Chandran B (2004). Concurrent expression of latent and a limited number of lytic genes with immune modulation and antiapoptotic function by Kaposi’s sarcoma-associated herpesvirus early during infection of primary endothelial and fibroblast cells and subsequent decline of lytic gene expression. J Virol.

[32] Bandyopadhyay C, Valiya-Veettil M, Dutta D, Chakraborty S, Chandran B (2014). CIB1 synergizes with EphrinA2 to regulate Kaposi’s sarcoma-associated herpesvirus macropinocytic entry in human microvascular dermal endothelial cells. PLoS Pathog.

[33] Chakraborty S, Veettil MV, Chandran B (2012). Kaposi’s sarcoma associated herpesvirus entry into target cells. Front Microbiol.

[34] Grove J, Marsh M (2011). The cell biology of receptor-mediated virus entry. J Cell Biol.

[35] Akula SM, Pramod NP, Wang FZ, Chandran B (2001). Human herpesvirus 8 envelope-associated glycoprotein B interacts with heparan sulfate-like moieties. Virology.

[36] Yu GY, Lai MM (2005). The ubiquitin-proteasome system facilitates the transfer of murine coronavirus from endosome to cytoplasm during virus entry. J Virol.

[37] Yang JY, Zong CS, Xia W, Wei Y, Ali-Seyed M, Li Z (2006). MDM2 promotes cell motility and invasiveness by regulating E-cadherin degradation. Mol Cell Biol.

[38] Greiner A, Lautwein A, Overkleeft HS, Weber E, Driessen C (2003). Activity and subcellular distribution of cathepsins in primary human monocytes. J Leukoc Biol.

[39] Schornberg KL, Shoemaker CJ, Dube D, Abshire MY, Delos SE, Bouton AH (2009). α5β1-integrin controls ebolavirus entry by regulating endosomal cathepsins. Proc Natl Acad Sci USA.

[40] Akula SM, Ford PW, Whitman AG, Hamden KE, Bryan BA, Cook PP (2005). B-Raf-dependent expression of vascular endothelial growth factor-A in Kaposi sarcoma-associated herpesvirus-infected human B cells. Blood.

[41] Grange PA, Gressier L, Williams JF, Dyson OF, Akula SM, Dupin N (2012). Cloning a human saliva-derived peptide for preventing KSHV transmission. J Invest Dermatol.

[42] Tang Q, Qin D, Lv Z, Zhu X, Ma X, Yan Q (2012). Herpes simplex virus type 2 triggers reactivation of Kaposi’s sarcoma-associated herpesvirus from latency and collaborates with HIV-1 Tat. PLoS One.

